# Informative prior on structural equation modelling with non-homogenous error structure

**DOI:** 10.12688/f1000research.108886.1

**Published:** 2022-05-04

**Authors:** Oladapo A. Olalude, Bernard O. Muse, Oluwayemisi O. Alaba

**Affiliations:** 1 Department of Statistics, University of Ibadan, Ibadan, Oyo State, +234, Nigeria; 2 Department of Mathematics and Statistics, Rufus Giwa Polytechnic, Owo, Ondo State, Nigeria

**Keywords:** Bayesian SEM, Latent Variable, Observed Variable, Heteroscedastic error structure, Predictive Performance

## Abstract

**Introduction: **This study investigates the impact of informative prior on Bayesian structural equation model (BSEM) with heteroscedastic error structure. A major drawback of homogeneous error structure is that, in most studies the underlying assumption of equal variance across observation is often unrealistic, hence the need to consider the non-homogenous error structure.

**Methods: **Updating appropriate informative prior, four different forms of heteroscedastic error structures were considered at sample sizes 50, 100, 200 and 500.

**Results:** The results show that both posterior predictive probability (PPP) and log likelihood are influenced by the sample size and the prior information, hence the model with the linear form of error structure is the best.

**Conclusions: **The study has been able to address sufficiently the problem of heteroscedasticity of known form using four different heteroscedastic conditions, the linear form outperformed other forms of heteroscedastic error structure thus can accommodate any form of data that violates the homogenous variance assumption by updating appropriate informative prior. Thus, this approach provides an alternative approach to the existing classical method which depends solely on the sample information.

## Introduction

Bayesian structural equation modeling (BSEM) analyses the relationship between the observed, unobserved, and latent variables within the Bayesian context. ^14^^,^^16^^,^^21^^,^^24^ The data visualization can be done by path diagram. In Bayesian inference, θ is random, which depicts the level of uncertainty about the true value of θ because both the observed data y and the parameters θ are assumed random. The joint probability of the parameters and the data as functions of the conditional distribution of the data given the parameters, and the prior distribution of the parameters can be modelled. More formally,

$$p(\theta \left|Y)\propto \right.p\left(\theta \right)p(Y\left|\theta )\right.$$ (1)

where

*P*( *θ|y*) is the posterior distribution

*P*( *θ*) is the prior distribution

*P*( *y|θ*) is the likelihood function

The un-normalized posterior distribution when expressed in terms of the unknown parameters *θ* for fixed values of y , this term is the likelihood *L*( *θ*| *y*). Thus, can be rewritten as:

$$p(\theta \left|Y)\propto \right.p\left(\theta \right)L(\theta \left|y)\right.$$ (2)

Studies abound on classical methods and Bayesian methods with a focus on homogeneous variance. ^8^^,^^19^^,^^22^^,^^25^ This study explores the BSEM using different forms of heteroscedastic error structure.

## Methods

### Bayesian estimation of structural equation models (SEM)

This section develops a Gibbs sampler to estimate SEM with reflective measurement indicators. ^1^^,^^11^^,^^12^ The Bayesian estimation is illustrated by considering a SEM that is equivalent to the mostly used model. A SEM is composed of a measurement equation (3) and a structural equation (4) ^9^:

$$y_{i}=\Lambda \omega_{i}+\varepsilon_{i}$$ (3)

$$\eta_{i}=\Pi \eta_{i}+\Gamma \xi_{i}+\delta_{i}$$ (4)

where $i\;\epsilon \;\left\{1,\ldots ,n\right\}$

It is assumed that measurement errors are uncorrelated with ω and δ , residuals are uncorrelated with ω and the variables are distributed as follows:

$$\varepsilon_{i}∼N\;\left(0,\Psi_{\varepsilon }\right)$$ (5)

$$\delta_{i}∼N\;\left(0,\Psi_{\delta }\right)$$ (6)

$$\omega_{i}∼N\;\left(0,\Sigma_{\omega }\right)$$ (7)

$\forall_{i}\;\epsilon \;\left\{1,\ldots ,n\right\}$ , where $\Psi_{\varepsilon }$ and $\Psi_{\delta }$ are diagonal matrices. The covariance matrix of ω is derived based on the SEM:

$$\Sigma_{\omega }=\left[\begin{array}{cc} E\;\left({\eta \eta }^{T}\right) & E\;\left({\xi \eta }^{T}\right)\\ E\;\left({\eta \xi }^{T}\right) & E\;\left({\xi \xi }^{T}\right) \end{array}\right]$$ (8)

$$\Sigma_{\omega }=\left[\begin{array}{cc} \Pi_{0}^{-1}\left({\Gamma \Phi \Gamma }^{T}+\Psi_{\delta }\right)\Pi_{0}^{-T} & \Pi_{0}^{-1}\Gamma \Phi \\ {\Phi \Gamma }^{T}\Pi_{0}^{-T} & \Phi \end{array}\right]$$ (9)

$${\eta \eta }^{T}=\left(\Pi_{0}^{-1}\Gamma \xi +\Pi_{0}^{-1}\delta \right){\left(\Pi_{0}^{-1}\Gamma \xi +\Pi_{0}^{-1}\delta \right)}^{T}=\Pi_{0}^{-1}\left({\Gamma \xi \xi }^{T}\Gamma^{T}+{\delta \delta }^{T}\right)\Pi_{0}^{-T}+\Pi_{0}^{-1}\left({\Gamma \xi \delta }^{T}+{\delta \xi }^{T}\Gamma^{T}\right)\Pi_{0}^{-T}E\;\left({\eta \eta }^{T}\right)=\Pi_{0}^{-1}\left({\Gamma \Phi \Gamma }^{T}+\Psi_{\delta }\right)\Pi_{0}^{-T}{\eta \xi }^{T}=\left(\Pi_{0}^{-1}\Gamma \xi +\Pi_{0}^{-1}\delta \right)\xi^{T}E\;\left({\eta \xi }^{T}\right)=\Pi_{0}^{-1}\Gamma$$ (10)

### Prior distributions

In order to enable Gibbs sampling from full conditional posterior distributions, natural conjugate prior distributions for the unknown parameters are considered. ^25^ Let $\psi_{\varepsilon \kappa }$ be the *k*th diagonal element of $\Psi_{\varepsilon }$ , $\psi_{\delta \iota }$ be the l th diagonal element of $\Psi_{\delta },\Lambda_{\kappa }^{T}$ be the *k*th row of Λ and $M_{\iota }^{T}$ be the *l*th row of *M*,

$$\psi_{\mathrm{\varepsilon k}}^{-1}∼\text{Gamma}\left(\alpha_{0\mathrm{\varepsilon k}},\beta_{0\mathrm{\varepsilon k}}\right)$$ (11)

$$\left[\Lambda_{k}|\psi_{\mathrm{\varepsilon k}}^{-1}\right]∼N\left(\Lambda_{0k},\psi_{\mathrm{\varepsilon k}},H_{0\Lambda k}\right)$$ (12)

$$\psi_{\mathrm{\delta i}}^{-1}∼\text{Gamma}\left(\alpha_{0\mathrm{\delta i}},\beta_{0\mathrm{\delta i}}\right)$$ (13)

$$\left[M_{i}|\psi_{\mathrm{\delta i}}^{-1}\right]∼N\left(M_{0i},\psi_{\mathrm{\delta i}},H_{0\mathrm{Mi}}\right)$$ (14)

$$\Phi ∼\mathrm{IW}\left[v_{0},V_{0}\right]$$ (15)

with $\kappa \;\epsilon \;\left\{1,\ldots ,p\right\}$ and $\iota \;\epsilon \;\left\{1,\ldots ,q1\right\}$

### Derivations of conditional distributions

The joint posterior of all unknown parameters is proportional to the likelihood times the prior,

$$p\;\left(\Lambda ,\Psi_{\varepsilon },\Omega ,M,\Psi_{\delta },\Phi |Y\right)\;\propto \;p\;\left(Y|\Lambda ,\Psi_{\varepsilon },\Omega ,M,\Psi_{\delta },\Phi \right)*p\;\left(\Lambda ,\Lambda_{\varepsilon },\Omega ,M,\Psi_{\delta },\Phi \right)$$ (16)

Given *Y* and Ω , Λ and $\Psi_{\varepsilon }$ are independent from $\Sigma_{\omega }$ . Draws of Ω , can cause estimation of Λ and $\Psi_{\varepsilon }$ as a simple regression model. Thus, sampling from the posterior distribution of Λ and $\Psi_{\varepsilon }$ without reference to $\Sigma_{\omega }.$ The same holds for inference with regard to *M*, Φ and $\Psi_{\delta }$ , which are independent from *Y* given Ω .

### Heteroscedastic error structures

The heteroscedastic error structure with different functional form of error variance under consideration are double logarithmic form, linear form, linear-inverse form and linear-absolute form as expressed in equation 17, 18, 19 and 20, respectively.

$$\sigma^{2}=\ln\sigma^{2}=\lambda_{o}^{*}+\lambda_{i}^{*}\ln\gamma_{i}^{*}+\mathrm{vi}$$ (17)

$$\sigma^{2}=|\varepsilon_{i}^{*}\varepsilon_{i}^{*'}|={\left(\lambda_{i}^{*}+\lambda_{2}^{*}\gamma_{i}^{*}+\mathrm{\nu i}\right)}^{2}$$ (18)

$$\sigma^{2}=|\varepsilon_{i}^{*}\varepsilon_{i}^{*'}|={\left(\lambda_{i}^{*}+\lambda_{2}^{*}\sqrt{\gamma_{i}^{*}}+\mathrm{\nu i}\right)}^{2}$$ (19)

$$\sigma^{2}=|\varepsilon_{i}^{*}\varepsilon_{i}^{*'}|={\left(\lambda_{i}^{*}+\lambda_{2}^{*}|\gamma_{i}^{*}|+\mathrm{\nu i}\right)}^{2}$$ (20)

Each of the functional forms of heteroscedastic error structure will be incorporated into the modified model. The variance matrix for disturbance vector is given as

$$\sum =\left(\varepsilon_{i}^{*}\varepsilon_{j}^{*'}\right)=\left({\sigma_{\lambda }^{2}}_{i}^{*},\;i=j\right)$$ (21)

$$\Omega =\left[\begin{array}{llll} \sigma^{2}\lambda_{i}^{*}\; & 0 & \cdots & 0\\ 0 & \sigma^{2}\lambda_{2}^{*}\; & \cdots & 0\\ \vdots & 0 & \cdots & 0\\ 0 & 0 & \cdots & \sigma^{2}\lambda_{n}^{*} \end{array}\right]$$ (22)

### The posterior distribution

The posterior density is the product of the likelihood and the prior distribution chosen ^2^^,^^13^

$$(P\left(\lambda^{*},\;h\right),\;\Omega |\;y^{*})\mathrm{\alpha p}\left(y^{*}|\lambda^{*},\;h,\;\Omega \right)p\left(\lambda^{*}\right)p\left(h\right)p\left(\Omega \right)$$ (23)

$$p\left(P\left(\lambda^{*},h\right),\Omega |y^{*}\right)=h\frac{N}{2}\left(\exp\left[-\frac{h}{2}{\left(\gamma^{*}-\lambda^{*}\right)}^{'}\left(\gamma^{*}-\lambda^{*}\gamma^{*}\right)\right]\right)\times h\frac{N+v-k\;}{2}\exp\left[-\frac{\mathrm{hv}}{2s^{-2}}\right]\times p\left(\Omega \right)\times \exp\left[-\frac{1}{2}{\left(\lambda^{*}-\lambda_{0}^{*}\right)}^{\prime }\right]\underline{V^{-1}}\left(\lambda^{*}-\lambda_{0}^{*}\right)$$ (24)

$$P\left(\lambda^{*},\;h,\;\Omega |y^{*}\right)=h\frac{N}{2}|\Omega |\frac{1}{2}\;\exp\;\left[-\frac{h}{2}\left(y^{*}-\lambda^{*}\gamma \right)\;\Omega^{-1}\;\left(y^{*}-\lambda^{*}\gamma \right)\right]\times \exp\left[-\frac{\underline{v^{-1}}}{2}\left({\underline{\lambda }}^{*}\;-\;\lambda_{0}^{*}\right)\;\underline{V^{-1}}\left({\underline{\lambda }}^{*}-\;\lambda_{0}^{*}\right)\right]\times n^{-1\left(\alpha +1\right)}\exp\left(\frac{-\beta }{h}\right)\times {\left|\Omega^{*}\right|}^{-1\left(\beta_{0}+k+1\right)/2}e^{-\mathrm{tr}\left(R_{0}^{-1}\beta_{0}^{-1}\right)/2}$$ (25)

Since the full posterior distribution is intractable; a Markov chain Monte Carlo (MCMC) simulation method of Gibbs sampling is employed. ^25^ This involves the use of marginal posterior distribution.

$$\lambda =\lambda_{0}^{*}={\left(\gamma \prime \;\Omega^{-1}\;\gamma \right)}^{-1}\gamma '\Omega^{-1}\gamma \;={\left(\gamma^{*'}\gamma \right)}^{-1}\gamma^{*'}\gamma^{*}$$ (26)

$$S^{2}=\frac{\left(\gamma^{*}\;-\;\gamma^{*}\;\lambda_{0}\right)\left(\gamma^{*}\;-\;\gamma^{*}\;\lambda_{0}\right)}{\underline{V}}$$

Also

$$\underline{{\mathrm{Vs}}^{2}}\;+\;\left(\lambda^{*}-\;{\hat{\lambda }}_{0}\right)'\;{{\gamma_{i}}^{*}}^{'}{\gamma_{i}}^{*}\;\left(\lambda^{*}\;-\;{\hat{\lambda }}_{0}\right)\;=\;\left(\gamma^{*}\;-\;{\gamma_{i}}^{*}\lambda^{*}\right)'\;\left(\gamma^{*}\;-\;{\gamma_{i}}^{*}\lambda^{*}\right)\;$$

$$p\left({\gamma_{i}}^{*}\;|\;,\gamma_{i},\;\sigma^{2}\right)\;=\;\frac{h^{v\;+k/2}}{\left(2\pi \right)\;\frac{N}{2}}\exp(-\frac{h}{2}\left(\underline{v}S^{2}\;+\;\left(\lambda^{*}\;-\;{\hat{\lambda }}_{0}\right)'\;{\gamma_{i}}^{'}\;{\gamma_{i}}^{*}\left(\lambda^{*}\;-\;{\hat{\lambda }}_{0}\right)\right)\;$$ (27)

$$\underline{v}\;=\;N-K\;\text{and}\;N\;=\;\underline{v}\;+\;K$$

Consider an informative prior created by set.

$$\underline{v^{-1}}\;j\;=\left(\frac{1}{c^{k}j}\right)\;\gamma_{j}^{*}\gamma_{j}^{*}$$

And letting *c* →0forj=1,2

The posterior distribution of $\lambda^{*}$ conditional on $\gamma^{*}$ , *h*, Ω is given by:

$$p\left(\lambda^{*}\;|\gamma^{*},\;h\;,\;\Omega \right)\;\alpha \;h^{\frac{N}{2}}\;\exp\;(-\frac{h}{2}\left(\gamma^{*}\;-\;\lambda^{*}\gamma^{*}\right)'\;\Omega^{-1}\left(\gamma^{*}\;-\;\lambda^{*}\;\gamma^{*}\right)\;+\;\left(\lambda^{*}\;-\;\lambda_{0}^{*}\right)'\;\underline{V^{-1}}\left(\gamma^{*}-\;\lambda_{0}^{*}\right)\times \exp\;\left[-\frac{h}{2\left(\Omega \right)}\left(\gamma_{i}^{*}\;-\;\lambda^{*}\;\gamma_{i}^{*}\right)'\;\left(\gamma_{i}^{*}\;-\;\lambda^{*}\;\gamma_{i}^{*}\right)\;+\frac{\left(\lambda^{*}\;-\;\lambda_{0}^{*}\right)'\;\left(\lambda^{*}\;-\;\lambda_{0}^{*}\right)}{\underline{v}}\right]$$ (28)

Solving the exponential part of the above equation, we will have:

$$\left(\gamma_{i}^{*}\;-\;\lambda^{*}\;\gamma_{i}^{*}\right)'\;\left(\gamma_{i}^{*\;}-\;\lambda^{*}\gamma_{i}^{*}\right)={\left(\gamma^{*}\right)}^{2}\;+\;{\left(\lambda^{*}\gamma^{*}\right)}^{2}\;-\;2y_{i}^{*}\;\lambda^{*}\;\gamma^{*}\;\text{and}\;\left(\gamma^{*}-\;\lambda_{0}^{*}\right)'\;\left(\lambda^{*}\;-\;\lambda_{0}^{*}\right)={\left(\lambda^{*}\right)}^{2}+\left({\left(\lambda_{0}^{*}\right)}^{2},-,2,\lambda^{*\;},\lambda_{0}^{*}\right)$$

Therefore,

$$=\;\exp\left[-\frac{h}{2\left(\Omega \right)}\sum_{i\;=1}^{N}y_{i}^{{*}^{2}}\;+\;{\left(\lambda^{*}\;\gamma^{*}\right)}^{2}-2y_{i}^{*}\lambda^{*}\gamma_{i}^{*}+{\frac{\left(\lambda^{*}-\lambda_{0}^{*}\right)}{\underline{v}}}^{2}\right]$$

The additional term not involving $\lambda^{*}$ is factored out to give:

$$=\exp\left[-\frac{\lambda^{*2}}{2\underline{v^{2}}}\;+\;\frac{\lambda^{*2}\lambda_{0}^{*}}{\underline{v^{2}}}+\;\frac{\lambda^{*}{\mathrm{ny}}^{*}}{\Omega \sigma^{2}}\;-\;\frac{n\lambda^{*2}}{2\Omega \sigma^{2\;}}\right]$$ (29)

Factorization in terms of $\lambda^{*}$ , the term in the exponential becomes:

$$=\;-\frac{\lambda^{*2}}{\sigma^{2}}+\frac{2{\lambda \lambda }_{*}^{*}}{2{\sigma_{*}}^{2}}\;$$

$$\sigma_{*}^{2}={\left(\frac{1}{\underline{v}}\;+\;\frac{n}{\Omega \sigma^{2}}\right)}^{-1}\;\text{and}\;\lambda_{*}^{*}\;=\sigma_{*}^{2}\left(\frac{\lambda_{0}^{2}}{\underline{v}}\;+\;\frac{{\mathrm{ny}}^{*}}{\Omega \sigma^{2}}\right)\;$$

So, the posterior density of $\lambda^{*}$ conditioned on other parameter *h*, Ω , *y* ^∗^ is a multivariate normal with mean $\lambda^{*}$ and variance $\sigma_{*}^{2}$ .

That is,

$$p\left(\lambda^{*}|\;h,\;\Omega ,y^{*}\right)∼N\left(\lambda_{*}^{*},\;\sigma_{*}^{2}\right)$$

The posterior distribution of *h* conditional on $\lambda^{*}$ , Ω , $y^{*}$ is given by:

$$P\left(\lambda^{*}|h,\;\Omega ,\gamma^{*}\right)\;\alpha \;h\frac{N}{2}\exp\left[-\frac{h}{2}\left(\gamma^{*}-\lambda^{*}\gamma^{*}\right)'\Omega^{-1}\left(\gamma^{*}-\lambda^{*}\gamma^{*}\right)\right.\times h\frac{N+v-k}{2}\;\left.\exp\left(-\frac{\mathrm{hv}}{2S^{2}}\right)\right]=h\frac{N+v-k}{2}\exp\left(-\frac{h}{2\Omega }\right)\sum_{i}^{N}\left(y_{i}^{2*},\;,+,n,{\left(\lambda^{*}\gamma^{*}\right)}^{2},-,2,y^{*},n,\lambda^{*\;},\gamma^{*},-,\;,\left[\frac{\mathrm{hv}}{2s^{2}}\right]\right)$$ (30)

The posterior distribution of Ω ^*^, conditional on *y* ^*^, λ ^*^, *h*, is given by:

$$P\left(\Omega |y^{*},\lambda^{*},h\right)\;\alpha P\left(\Omega \right)\;\times \;h\frac{N}{2}\;\exp\left[-\frac{h}{2}\left(y^{*}-\lambda^{*}\gamma^{*}\right)\prime \Omega^{-1}\left(y^{*}-\lambda^{*}\gamma^{*}\right)\right]$$ (31)

$$P\left(\Omega |y^{*},\lambda^{*},h\right)\;\alpha h\frac{N}{2}\;\exp\left[-\frac{h}{2}\left(y^{*}-\lambda^{*}\gamma^{*}\right)\prime \Omega^{-1}\left(y^{*}-\lambda^{*}\gamma^{*}\right)\right]\;\times {\left|\Omega^{*}\right|}^{-(\beta_{0}+k+1)/2}\;\exp\;\mathrm{tr}\left(R_{0}^{-1}\beta_{0}^{-1}\right)/2$$ (32)

### The Gibbs sampler

The Gibbs sampling procedure used in this study involves generation of sequence of draws from the conditional posterior distribution of each parameter. ^2^^,^^22^^,^^25^

### Gibbs sampling procedure

(i) Chose a starting or initial value, $\phi^{\left(0\right)}$ for s=1,2,…,S

(ii) Take a random draw, $\phi_{1}^{x}$ from the full conditional, $p\left(\phi_{\left(1\right)}|y,\phi_{\left(1\right)}^{\left(x-1\right)}\right)$

(iii) Take a random draw, $\phi_{2}^{x}$ from the full conditional, $p\left(\phi_{\left(2\right)}|y,\phi_{\left(1\right)}^{\left(x\right)}\right)$ using the updated values of $\phi_{1}^{x}$

(iv) Repeat until *M* draws are obtained, each being a vector of $\phi^{\left(x\right)}$

(v) Perform the Burn-in by dropping the first $S_{\left(0\right)}$ of these draws to eliminate the effect of $\phi_{0}$ , the remaining $S_{1}$ draws are then averaged to obtain the estimate of the posterior $E\left[g\left(\phi \right)/y\right]$ .

The right-hand side of ( 15) is proportional to the density function of an inverse Wishart distribution

Then,

$$P\;\left(\Phi |Y,\Omega \right)∼{\mathrm{IW}}_{q}\;\left[\left({\Omega \Omega }^{T}R_{0}^{-1}\right),n+\rho_{0}\right]$$ (33)

### Design of simulation

• At different functional forms of ^3^ heteroscedastic error structure with changes in sample size of 50, 100, 200 and 500. Hyper-parameter will be arbitrarily chosen for the simulation using Gibbs sampler an MCMC method. ^6^^,^^22^
• The R code can be accessed via the *Extended data.* ^26^
• Factor loading and error precision followed multivariate normal and inverse gamma distributions respectively to assess the prior sensitivity. ^21^
• The criteria that will be used to assess the performance of the posterior simulation technique are the posterior estimates.

In order to evaluate the Bayesian model fit, we used the posterior predictive probability (PPP) procedure. ^4^^,^^5^^,^^7^^,^^24^

$$\mathrm{PPP}=P(f\left(y,\hat{\lambda }i\right)<f\left(y^{\mathrm{rep}},{\hat{\lambda }}_{i}\right)\equiv \frac{1}{m}\;\sum_{i=1}^{m}\delta_{i}$$ (34)

After achieving convergence (after *j* iterations). $\left({\hat{\lambda }}^{*\left(j+1\right)},\lambda^{\left(j+1\right)},\Omega^{\left(j+1\right)}\right)$ can be regarded as observation from *p*(λ*, Ω| *y*) collect $\left[\left(\lambda^{*\left(t\right)},\Omega^{*\left(t\right)}\right)\;t=j+1,.\ldots ,+T\right]$ for statistical inference.

$$\hat{\lambda }=T^{-1}\sum_{t=1}^{T}\lambda^{\left(t\right)},\hat{\Omega }\;=T^{-1}\;\sum_{t=1}^{T}\Omega^{\left(t\right)}$$ (35)

gives Bayesian estimates of parameter and the latent variables. ^10^^,^^17^^,^^23^

## Results and discussion

The section presents the discussion of analysis of results; performances of the estimators across the parameters for the different forms of heteroscedasticity, performances of Bayesian posterior simulation and analytical methods in the presence of heteroscedasticity via consideration of four (4) different forms of heteroscedastic error structures over four sample sizes of 50, 100, 200 and 500.

### Performance of the estimators at heteroscedasticity condition

This gives the results for the latent and observed variables at various sample sizes for the four heteroscedastic error conditions considered.

### Comparison of latent variable estimates at different sample sizes under the heteroscedasticity condition

Using the assumed values for the estimates which are $\lambda_{1}$ = 2.0, $\lambda_{2}$ = 3.0 and precision = 15.0.

The covariance matrix of *ω* was derived to be $E\cdot \mathrm{\eta \xi T}=\prod_{0}^{-1}\Gamma$ with *M* at fixed values (0 or 1). The Bayesian estimates of SEM using the independent normal-gamma priors were derived for the two classes of SEM. Hyper-parameter was arbitrarily chosen for the simulation using Gibbs sampler a Markov chain Monte Carlo (MCMC) method since the joint posterior density does not have a tractable form. For the double logarithmic form, at 95% credible interval, when n=50, Posterior Mean, PM, and Precision, PR (2.011, 2.435, and 13.202), Posterior Standard Deviation PSD (0.035, 0.033, and 0.223) and when n=100, PM, and PR (2.022, 2.528, and 13.70), PSD (0.023, 0.025, and 0.251), when n=200, PM, and PR (2.052, 2.611, and 14.4), PSD (0.017, 0.018, and 0.255), when n=500, PM, and PR (2.010, 2.801, and 14.7), PSD (0.031, 0.021, and 0.258).

For the linear form, when n=50, PM, and PR (1.845, 2.779, and 13.95), PSD (0.240, 0.242, and 0.235). When n=100, PM, and PR (1.861, 2.811, and 14.22), PSD (0.328, 0.226, and 0.325), when n= 200, PM, and PR (1.956, 2.921, and 14.72), PSD (0.219, 0.217, and 0.212), and when n=500, PM, and PR (2.120, 3.122, and 14.95), PSD (0.211, 0.311, and 0.114).

For the linear-inverse form when n=50, PM, and PR (1.882, 2.742, and 14.95), PSD (0.040, 0.028, and 0.291). When n=100, PM, and PR (1.972, 2.835, and 14.65), PSD (0.024, 0.023, and 0.229). When n=200, PM, and PR (1.988, 2.901, and 14.45), PSD (0.017, 0.016, and 0.109), and when n=500, PM, and PR (2.021, 3.003, and 14.21), PSD (0.011, 0.015, and 0.105).

For the linear-absolute form, when n=50, PM, and PR (2.036, 2.824, and 14.500), PSD (0.032, 0.034, and 0.122), When n=100, PM, and PR (1.908, 2.903, and 13.92), PSD (0.022, 0.026, and 0.234). When n=200, PM, and PR (1.893, 2.809, and 13.85), PSD (0.017, 0.023, and 0.311), and when n=500, PM, and PR (1.806, 2.788, and 13.55), PSD (0.031, 0.035, and 0.433).

Examining different forms of heteroscedastic error structures in Bayesian structural equation modeling using informative priors, rather than assuming homogenous variance which is often a statistical fallacy in many studies. We compare the models’ posterior means and standard deviations in Tables 1, 2, 3 and 4. The differences are unlikely to impact substantive conclusions, but two of them are noteworthy.

**Table 1.** Double logarithmic form on latent variable and observed variable estimates.

Sample sizes	Latent variables	Posterior Mean (PM)	Posterior Standard Deviation (PSD)	Credible Interval (CI)		Measured variables	Estimate	Standard Deviation
**n=50**	$\lambda_{1}$	2.011	0.035	1.959	2.062	*x*_1_	0.045	0.023
	$\lambda_{2}$	2.435	0.033	2.384	2.485	*x*_2_	0.038	0.023
	Precision (PR)	13.202	0.223	13.071	13.332			
**n=100**	$\lambda_{1}$	2.022	0.023	1.979	2.064	*x*_1_	0.053	0.008
	$\lambda_{2}$	2.528	0.025	2.484	2.571	*x*_2_	0.037	0.024
	Precision	13.700	0.251	13.561	13.838			
**N=200**	$\lambda_{1}$	2.052	0.017	2.015	2.088	*x*_1_	0.006	0.045
	$\lambda_{2}$	2.611	0.018	2.573	2.648	*x*_2_	0.048	0.020
	Precision	14.4	0.255	14.260	14.539			
**N=500**	$\lambda_{1}$	2.010	0.031	1.961	2.058	*x*_1_	0.040	0.028
	$\lambda_{2}$	2.801	0.021	2.760	2.841	*x*_2_	0.018	0.004
	Precision	14.7	0.258	14.559	14.840			

**Table 2.** Linear form on latent variable and observed variable estimates.

Sample sizes	Latent variables	Posterior Mean (PM)	Posterior Standard Deviation (PSD)	Credible Interval (CI)		Measured variables	Estimate	Standard Deviation
**n=50**	$\lambda_{1}$	1.845	0.240	1.709	1.981	*x*_1_	0.078	0.017
	$\lambda_{2}$	2.779	0.242	2.643	2.915	*x*_2_	0.055	0.036
	Precision	13.950	0.235	13.816	14.844			
**n=100**	$\lambda_{1}$	1.861	0.328	1.702	2.0197	*x*_1_	0.079	0.012
	$\lambda_{2}$	2.811	0.226	2.679	2.943	*x*_2_	0.036	0.028
	Precision	14.220	0.325	14.062	14.378			
**N=200**	$\lambda_{1}$	1.956	0.219	1.826	2.086	*x*_1_	0.071	0.008
	$\lambda_{2}$	2.921	0.217	2.792	3.050	*x*_2_	0.047	0.016
	Precision	14.72	0.212	14.542	14.898			
**N=500**	$\lambda_{1}$	2.120	0.211	1.993	2.247	*x*_1_	0.052	0.022
	$\lambda_{2}$	3.122	0.311	2.967	3.277	*x*_2_	0.059	0.010
	Precision	14.95	0.114	14.857	15.044			

**Table 3.** Linear inverse form on latent variable and observed variable estimates.

Sample sizes	Latent variables	Posterior Mean (PM)	Posterior Standard Deviation (PSD)	Credible Interval (CI)		Measured variables	Estimate	Standard Deviation
**n=50**	$\lambda_{1}$	1.882	0.043	1.827	1.937	*x*_1_	0.075	0.020
	$\lambda_{2}$	2.742	0.028	2.696	2.788	*x*_2_	0.023	0.017
	Precision	14.95	0.291	14.801	15.099			
**n=100**	$\lambda_{1}$	1.972	0.024	1.929	2.015	*x*_1_	0.055	0.010
	$\lambda_{2}$	2.835	0.023	2.793	2.877	*x*_2_	0.031	0.021
	Precision	14.65	0.229	14.317	14.583			
**N=200**	$\lambda_{1}$	1.988	0.017	1.826	2.102	*x*_1_	0.054	0.006
	$\lambda_{2}$	2.901	0.016	2.790	3.012	*x*_2_	0.032	0.024
	Precision	14.45	0.109	14.358	14.541			
**N=500**	$\lambda_{1}$	2.021	0.011	1.992	2.050	*x*_1_	0.052	0.015
	$\lambda_{2}$	3.003	0.015	2.969	3.037	*x*_2_	0.050	0.022
	Precision	14.210	0.105	14.120	14.300			

**Table 4.** Linear absolute form on latent variable and observed variable estimates.

Sample sizes	Latent variables	Posterior Mean (PM)	Posterior Standard Deviation (PSD)	Credible Interval (CI)		Measured variables	Estimate	Standard Deviation
**n=50**	$\lambda_{1}$	2.036	0.032	1.986	2.086	*x*_1_	0.043	0.018
	$\lambda_{2}$	2.824	0.034	2.773	2.875	*x*_2_	0.027	0.022
	Precision	14.500	0.122	14.403	14.597			
**n=100**	$\lambda_{1}$	1.908	0.022	1.867	1.949	*x*_1_	0.047	0.017
	$\lambda_{2}$	2.903	0.026	2.858	2.948	*x*_2_	0.043	0.025
	Precision	13.92	0.234	13.786	14.054			
**N=200**	$\lambda_{1}$	1.893	0.017	1.857	1.929	*x*_1_	0.054	0.017
	$\lambda_{2}$	2.809	0.023	2.767	2.851	*x*_2_	0.041	0.024
	Precision	13.85	0.311	13.696	14.005			
**N=500**	$\lambda_{1}$	1.806	0.031	1.757	1.855	*x*_1_	0.048	0.019
	$\lambda_{2}$	2.788	0.035	2.736	2.840	*x*_2_	0.044	0.022
	Precision	13.55	0.433	13.367	13.732			

**Figure 1.**
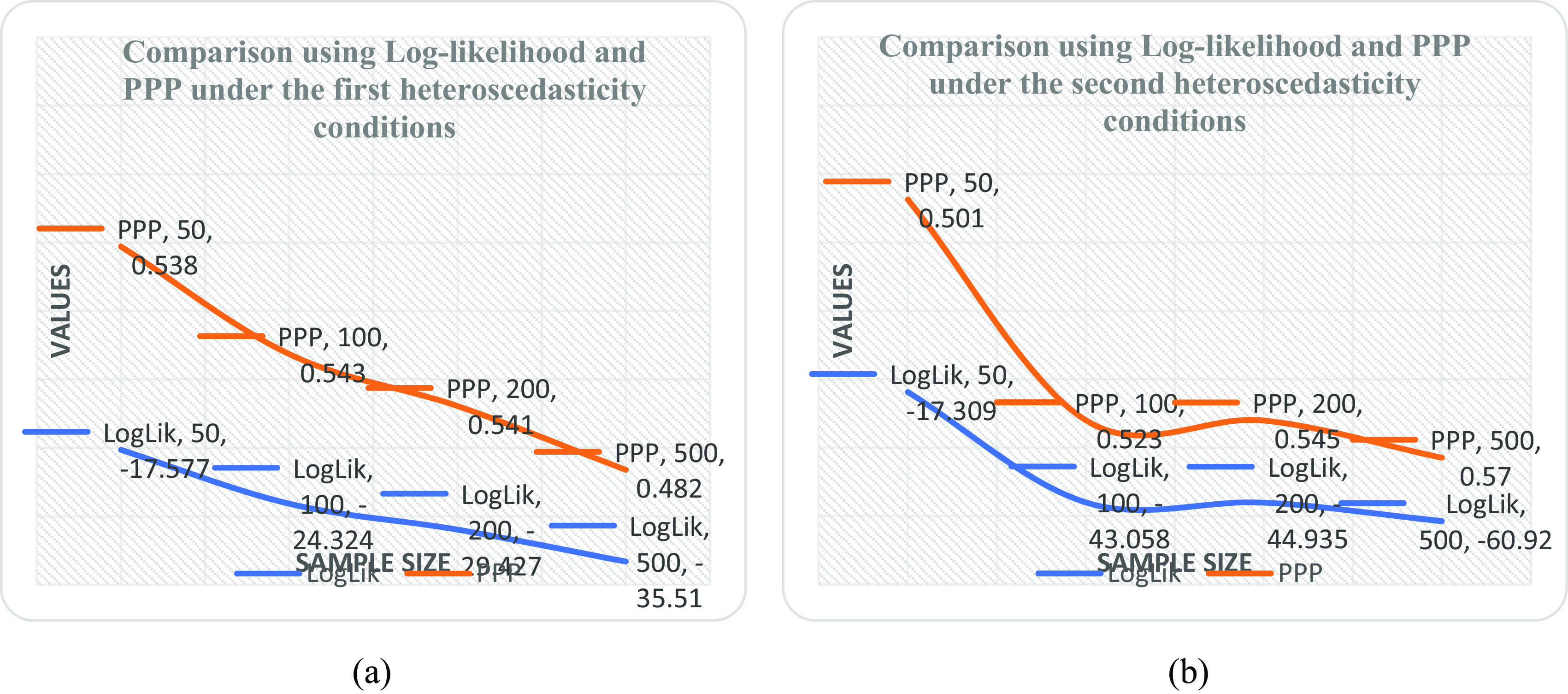
Plot of log likelihood and posterior predictive probability (PPP) at various sample sizes under (a) the double logarithmic form and (b) the linear form.

**Figure 2.**
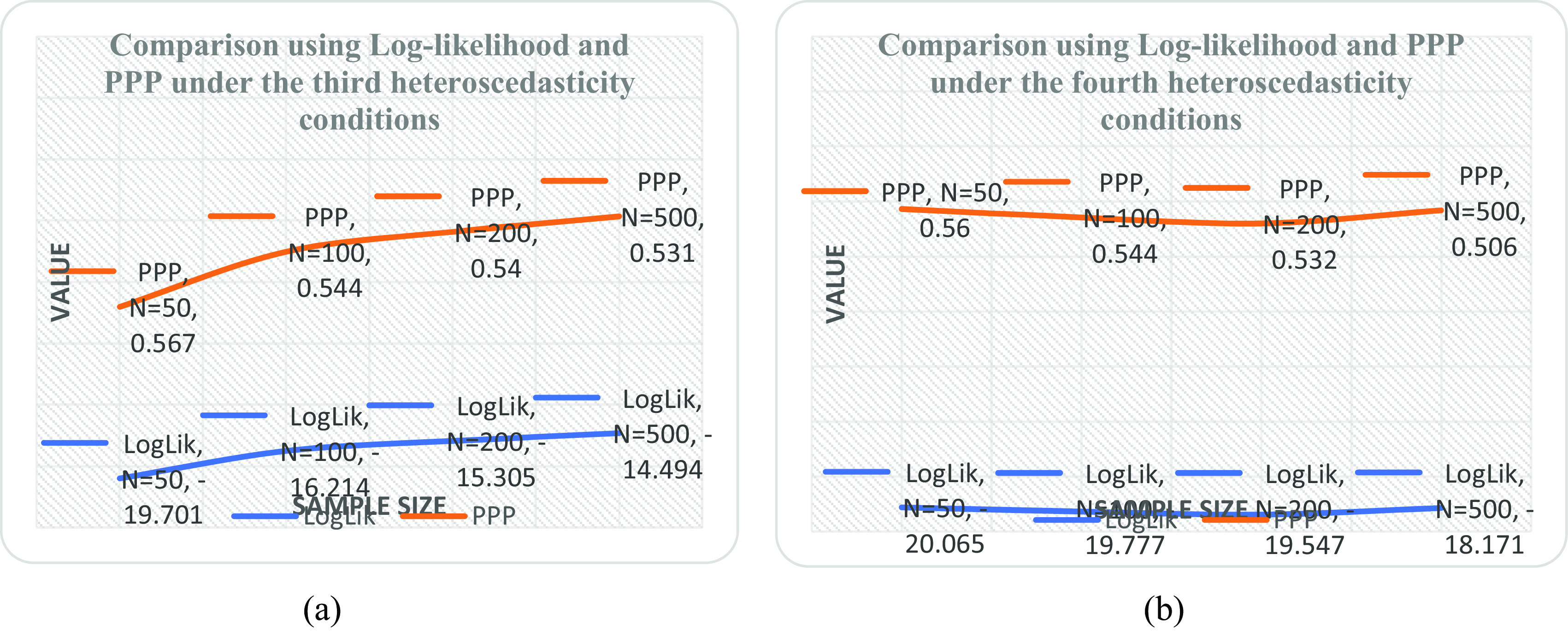
Plot of log likelihood and posterior predictive distribution (PPP) at various sample sizes under (a) the linear-inverse form (b) the linear-absolute form.

First, the posterior means of the loadings ( $\lambda_{1}$ and $\lambda_{2}$ ) are somewhat smaller under different heteroscedastic condition with the informative priors as observed in Tables 6 and 7. Second, the factor variance $\gamma^{*}\;$ is larger under our model with informative priors, likely because the informative prior placed more density on larger values of the posterior standard deviation. An evaluation of the model fit was based on the values of PPP as shown in Table 5 and it was observed that the linear form is the best with minimum PPP value as sample size increases. It was also revealed by the downward slope of the model as the sample size increases from 50 to 500 shown in Figure 1b when compared with Figure 1a, 2a and 2b.

**Table 5.** Comparison at varying sample sizes of different heteroscedastic form.

Sample size	Double logarithmic		Linear		Linear inverse		Linear absolute	
	LogLik	PPP	LogLik	PPP	LogLik	PPP	LogLik	PPP
N=50	-17.577	0.538	-17.309	0.501	-19.701	0.567	-20.065	0.560
N=100	-24.324	0.543	-43.058	0.523	-16.214	0.544	-19.777	0.544
N=200	-29.427	0.541	-44.935	0.545	-15.305	0.540	-19.547	0.532
N=500	-35.510	0.482	-60.920	0.570	-14.494	0.531	-18.171	0.506

**Table 6.** Latent variable estimates at different sample sizes under the double-logarithmic and linear forms.

Sample size	Latent variables	Double logarithmic				Linear			
		Posterior Mean (PM)	Posterior Standard Deviation (PSD)	Credible Interval (CI)		Posterior Mean (PM)	Posterior Standard Deviation (PSD)	Credible Interval (CI)	
N=50	$\lambda_{1}$	2.001	0.231	1.868	2.134	2.110	0.230	1.977	2.243
	$\lambda_{2}$	2.283	0.538	2.080	2.486	2.554	0.201	2.430	2.678
N=100	$\lambda_{1}$	2.021	0.312	1.866	2.176	2.020	0.123	1.923	2.117
	$\lambda_{2}$	2.478	0.562	2.270	2.686	2.601	0.356	2.436	2.766
N=200	$\lambda_{1}$	2.032	0.432	1.850	2.214	2.011	0.174	1.895	2.127
	$\lambda_{2}$	2.770	0.832	2.517	3.023	2.705	0.456	2.518	2.892
N=500	$\lambda_{1}$	2.100	0.445	1.915	2.285	2.005	0.253	1.866	2.144
	$\lambda_{2}$	2.888	1.564	2.541	3.234	3.102	0.575	2.892	3.312

Note: Posterior mean (PM), posterior standard deviation (PSD), credible interval (CI).

**Table 7.** Latent variable estimates at different sample sizes under the linear-inverse and linear absolute forms.

Sample size	Latent variables	Linear-inverse				Linear-absolute			
		Posterior Mean (PM)	Posterior Standard Deviation (PSD)	Credible Interval (CI)		Posterior Mean (PM)	Posterior Standard Deviation (PSD)	Credible Interval (CI)	
N=50	$\lambda_{1}$	2.101	0.352	1.937	2.265	1.732	0.311	1.577	1.887
	$\lambda_{2}$	2.637	0.528	2.436	2.838	2.582	0.583	2.370	2.794
N=100	$\lambda_{1}$	1.982	0.421	1.802	2.162	1.810	0.252	1.671	1.949
	$\lambda_{2}$	2.754	0.192	2.633	2.875	2.634	0.375	2.464	2.804
N=200	$\lambda_{1}$	1.975	0.476	1.784	2.166	1.820	0.211	1.696	1.947
	$\lambda_{2}$	2.814	0.901	2.551	3.077	2.723	0.766	2.480	2.966
N=500	$\lambda_{1}$	2.111	0.488	1.917	2.305	1.920	0.145	1.815	2.026
	$\lambda_{2}$	3.073	1.102	2.782	3.364	2.902	0.331	2.743	3.062

Considering an improvement to maximum likelihood method, in Bayesian estimations, parameters are considered as random with informative prior distribution also known as the conjugate family of the posterior, once the data is simulated/collected, it is combined with prior distribution using Bayes theorem, next posterior distribution is calculated reflecting the prior knowledge and simulated data. ^14^^,^^15^^,^^21^ Joint posterior distribution is summarized using MCMC simulation techniques in terms of lower dimensional summary statistics as posterior mean and posterior standard deviations. ^5^^,^^25^ We observe that the structural and measurement equation obtained from this study are adequate and in general we could accept the proposed model.

## Conclusion

In this research, the derived Bayesian estimators of a structural equation model in the presence of different forms of heteroscedastic error structures validated accurate statistical inference. The study has also been able to address sufficiently the problem of heteroscedasticity of known form using four different heteroscedastic conditions for both linear and quadratic forms, and it has also successfully modified the homogenous error structure to heteroscedastic error structure in Bayesian structural equation model. ^20^ The linear form outperformed other forms of heteroscedastic error structure thus can accommodate any form of data that violates the homogenous variance assumption by updating appropriate informative prior. ^16^^,^^18^ Thus, this approach provides an alternative approach to the existing classical method which depends solely on the sample information.

## Data availability

### Underlying data

All data underlying the results are available as part of the article and no additional source data are required.

### Extended data

Figshare: RCODE BSEM.docx. https://doi.org/10.6084/m9.figshare.19299851. ^26^

Data are available under the terms of the Creative Commons Zero “No rights reserved” data waiver (CC0 1.0 Public domain dedication).
