# Supplementary material for: Informative prior on structural equation modelling with non-homogenous error structure
Source: F1000Res. 2022 Sep 20;11:494. Originally published 2022 May 4. [Version 2] doi: 10.12688/f1000research.108886.2 (PMC9515605; doi:10.12688/f1000research.108886.2)
Supplement: Peer review history

# Peer review: Reviewer response for version 2

**DOI:** 10.5256/f1000research.137309.r150953

**Published:** 2022-09-27

**Authors:** Mohamed R. Abonazel

**Affiliations:** 1 Department of Applied Statistics and Econometrics, Faculty of Graduate Studies for Statistical Research, Cairo University, Giza, Egypt

I am happy with the corrections in the revised paper. It was improved. So, the current version of this manuscript is suitable for indexing. Good luck.

Is the work clearly and accurately presented and does it cite the current literature?

Partly

If applicable, is the statistical analysis and its interpretation appropriate?

Yes

Are all the source data underlying the results available to ensure full reproducibility?

Yes

Is the study design appropriate and is the work technically sound?

Yes

Are the conclusions drawn adequately supported by the results?

Yes

Are sufficient details of methods and analysis provided to allow replication by others?

Yes

Reviewer Expertise:

Econometrics, R Programming, Panel Data, Time Series, Computational Statistics,  Data Analysis, R Statistical Packages, Statistical Modeling,  Nonparametric Models, Robust Regression.

I confirm that I have read this submission and believe that I have an appropriate level of expertise to confirm that it is of an acceptable scientific standard.

# Peer review: Reviewer response for version 1

**DOI:** 10.5256/f1000research.120326.r136840

**Published:** 2022-07-26

**Authors:** Mohamed R. Abonazel

**Affiliations:** 1 Department of Applied Statistics and Econometrics, Faculty of Graduate Studies for Statistical Research, Cairo University, Giza, Egypt

This paper investigated the impact of informative prior on Bayesian structural equation model with heteroscedastic error structure. Four different forms of heteroscedastic error structures were considered. The suggested Bayesian approach provides an alternative approach to the existing classical method which depends solely on the sample information. The results indicate that the suggested Bayesian estimation method is more efficient than the existing classical method.

 In my opinion, the paper offers a good contribution. So, I recommend accepting this paper, but after making the following modifications to improve the manuscript:

1. I think the title of the paper needs improvement. I suggest the following title: “Bayesian estimation of structural equation modelling with non-homogenous error structure”.
2. In the “abstract” section, the findings or research results should be introduced briefly in the abstract. 
3. In the “introduction” section, the introduction did not contain enough background information. Also discuss the similar work that has been done in this area to give a detailed view of this work. The authors should add more papers related to the Bayesian estimation of structural equation modelling.
4. In the “methods” section, the authors should define each symbol given in each equation.
5. In the “conclusion” section, the limitation and future research directions should be mentioned.

Is the work clearly and accurately presented and does it cite the current literature?

Partly

If applicable, is the statistical analysis and its interpretation appropriate?

Yes

Are all the source data underlying the results available to ensure full reproducibility?

Yes

Is the study design appropriate and is the work technically sound?

Yes

Are the conclusions drawn adequately supported by the results?

Yes

Are sufficient details of methods and analysis provided to allow replication by others?

Yes

Reviewer Expertise:

Applied statistics, Econometric models.

I confirm that I have read this submission and believe that I have an appropriate level of expertise to confirm that it is of an acceptable scientific standard, however I have significant reservations, as outlined above.

# Peer review: Reviewer response for version 1

**DOI:** 10.5256/f1000research.120326.r140694

**Published:** 2022-07-12

**Authors:** Adenike Oluwafunmilola Olubiyi

**Affiliations:** 1 Department of Statistics, Ekiti State University, Ado Ekiti, Nigeria

**ABSTRACT**

1. In this paper, the research team investigates the impact of informative prior on Bayesian Structural equation model (BSEM) with heteroscedastic error structure.
2. The drawback of homogeneous error structure was addressed by considering the non-homogenous error structure.

**General Comment under this section**: the researcher can add their findings

**INTRODUCTION**

1. Bayesian structural equation modelling (BSEM) analyses the relationship between the observed, unobserved and latent variables within the Bayesian context
2. The likelihood is the un-normalized posterior distribution when expressed in terms of the unknown parameters θ for fixed values of y.
3. The study explores the BSEM using different forms of heteroscedastic error structure.
4. Other studies abound on classical methods and Bayesian methods with focus on homogeneous variance. ^8,19,22,25 ^

**General Comment under this section:** The Introductory part was well presented and detailed with relevant citation, even though the researchers can still explore more.

**Methods **

1. Gibbs sampler was developed to estimate SEM with reflective measurement indictors. ^1,11,12^
2. The SEM equation used is composed of a measurement equation and a structural equation. ^9^
3. To enable Gibbs sampling from full conditioner posterior distributions, natural conjugate prior distributions for the unknown parameters were considered.
4. The heteroscedastic error structure with different functional form of error variance under consideration are double logarithm form, linear form, linear-inverse form and linear-absolute form as expresses in equation 17,18,19 and 20.
5. Markov Chain Monte Carlo (MCMC) Simulation method of Gibbs Sampling was employed.

**SIMULATION**

1. At different functional forms of ^3^ heteroscedastic error structure with changes in sample size of 50,100,200 and 500. Hyper-parameter was arbitrarily chosen for the simulation using Gibbs sampler an MCMC method.
2. To assess the prior sensitivity, factor loading and error precision followed multivariate normal and inverse gamma distributions respectively.
3. The posterior estimate is used to assess the performance of the posterior simulation technique.
4. In order to evaluate the Bayesian Model fit, the researcher used the posterior predictive probability (PPP) procedure. ^4,5,7,24^

General Comment under this section: The methods were well presented and the simulation study well organized.

**RESULTS**

1. This gives the results for the latent and observed variables at various sample sizes for the four heteroscedastic error conditions considered using the assumed values for the estimates which are λ _1_ = 2.0, λ _2_ = 3.0 and precision 15.0.
2. The posterior means of loadings λ _1 _and λ _2_ are somewhat smaller under different heteroscedastic condition with the informative priors.
3. It was observed that the linear form is the best with minimum PPP value as sample size increases.
4. It was also revealed by the downward slope of the model as the sample size increases from 50 500.
5. It was observed that the structural and measurement equation obtained from this study are adequate and in general could be accepted for the proposed model.

**General comment under this section:** The obtained results in this section indicate the correct performances with increased sample sizes and the incorporation of informative priors.

**CONCLUSION**

1. The study has been able to address sufficiently the problem of heteroscedasticity of known form using four different heteroscedasticity of known form using four different heteroscedastic conditions for both linear and quadratic forms.
2. It has also successfully modified the homogenous error structure to heteroscedastic error structure in Bayesian structural equation model.
3. Thus, the approach provides an alternative approach to the existing classical method which depends solely on sample information.

**General comment:** this section flows with the contents of the paper and is well presented.

**The manuscript is well written and followed the format of the Journal and has substance; the manuscript can be approved.**

Is the work clearly and accurately presented and does it cite the current literature?

Yes

If applicable, is the statistical analysis and its interpretation appropriate?

Yes

Are all the source data underlying the results available to ensure full reproducibility?

Yes

Is the study design appropriate and is the work technically sound?

Yes

Are the conclusions drawn adequately supported by the results?

Yes

Are sufficient details of methods and analysis provided to allow replication by others?

Yes

Reviewer Expertise:

Environmental Statistics and Econometric

I confirm that I have read this submission and believe that I have an appropriate level of expertise to confirm that it is of an acceptable scientific standard.

## References

[1] : Bayesian factor analysis for multilevel binary observations. *Psychometrika* .2000;65(4) : 10.1007/BF02296339 475-496 10.1007/BF02296339

[2] : Some Methods for Respecifying Measurement Models to Obtain Unidimensional Construct Measurement. *Journal of Marketing Research* .1982;19(4) : 10.2307/3151719 10.2307/3151719

[3] : DYNAMIC PROGRAMMING AND LAGRANGE MULTIPLIERS. *Proc Natl Acad Sci U S A* .1956;42(10) : 10.1073/pnas.42.10.767 767-9 10.1073/pnas.42.10.767 16589948PMC528332
